# Enhancing facility maintenance using knowledge-based BIM systems

**DOI:** 10.1038/s41598-025-30057-7

**Published:** 2025-12-09

**Authors:** A. Ehab, Mazkour A. Mahdi, Arafa El-Helloty

**Affiliations:** 1 Civil Engineering Department, Badr University in Cairo (BUC), Cairo, 11829 Egypt; 2 Civil Engineering Department, Al-Azhar University, Cairo, Egypt

**Keywords:** Facility management, Decision support system, Building information modeling, Predictive maintenance, Engineering, Civil engineering

## Abstract

The operation phase of a building’s lifecycle refers to the period during which the building is in active use. So, to keep all these facilities working with acceptable performance, maintenance is necessary to keep the buildings working throughout their life cycle. Building maintenance and facility management are very complicated by traditional methods like personal estimation or manual inspections. On the other hand, the growth of technology, mainly in construction, helps in different fields of construction management. A big example of this technology is Building Information Modeling (BIM), a digital representation tool of buildings at various phases of the construction life cycle. To get more simplification, many commercial plugins for construction maintenance have been issued, which are integrated with the BIM model. Unfortunately, these plugins require specialized knowledge, are complicated, and need expertise, training, and more accuracy. In addition, the needed improvement in the maintenance process to overcome the challenges and obstacles that the organization is currently facing. This paper proposes a decision support system framework plugin to help facility management teams effectively plan, track, and allocate their maintenance budgets with a very simple, flexible, and easy-to-use system. The proposed framework creates maintenance schedules based on the facility’s condition, type of equipment, a frequency of use, and allocates the necessary resources, parts, and labor required for the maintenance phase. It can also generate reports that show resource utilization, expenditure, and other cost-related indicators, making it easier for facility managers to plan their budgets and make informed decisions. The main idea of the proposed framework plugin that was created using Python is to use real-time data and predictive models to optimize maintenance plans, schedules, and resource allocation, facility management teams. The results show reduced downtime, minimized costs, and extended the lifespan of building assets within an actual case study. This framework plugin is an exemplary approach for facilities seeking to implement an innovative yet scalable Facility management (FM) optimum solution.

## Introduction

A maintenance system is a plan or set of procedures that is put in place to help organizations manage their maintenance expenses. The impact of these systems (which are considered a part of facility management) has prompted researchers to address existing gaps in knowledge and improve various aspects of these systems. Despite the progress made, there are still gaps that current technologies cannot adequately address.

The emergence of smart and advanced manufacturing technologies has also posed new challenges to maintenance systems. Effectively addressing these gaps can significantly enhance the profitability throughout the building life cycle. Building Information Modeling (BIM) is one of these technologies that gained prominence in the past decade as a leading tool for managing the entire life cycle of buildings. The National Institute of Building Sciences defines it as a digital representation of a facility’s physical and functional characteristics.

A maintenance system with BIM is a software application or plugin designed to help expert managers and facility maintenance staff track and organize information related to the upkeep of a building. It’s complicated to help with tasks such as scheduling routine maintenance, tracking equipment warranties and service records, and organizing work orders and asset inventories^1^. One of the key benefits of a BIM maintenance system is that it can enable more efficient and effective maintenance management by providing real-time data on building operations and equipment performance^2^.

By leveraging this data, building managers can identify and address issues proactively, reducing downtime and maintenance costs over the long term. Overall, a BIM maintenance system can help building managers improve the longevity and performance of their building assets while providing a safer, more comfortable, and more productive environment for occupants^3^. Overall, BIM capabilities in operation and maintenance can enable expert facility managers to monitor and optimize building performance, leading to reduced energy consumption, lower operating costs, and higher occupancy comfort.

## Background

**Fig. 1:**
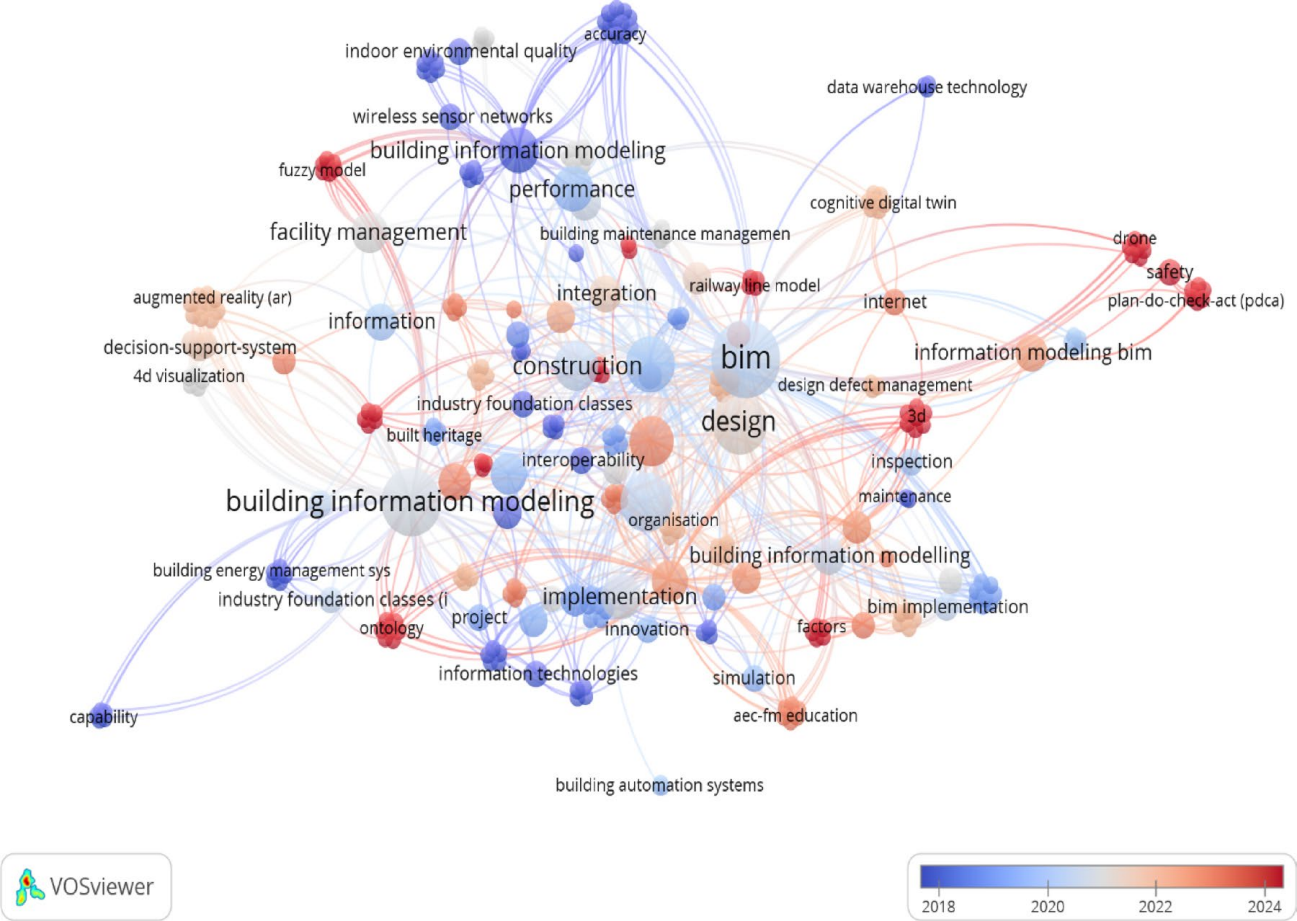
Cluster analysis.

Building Information Modeling (BIM) and Facility Management (FM) have transformed the construction industry by enhancing collaboration, improving project efficiency, and fostering innovation. So, to make this area under spot, a bibliometric analysis focuses on the co-occurrence of keywords BIM capabilities, building management, and predictive maintenance as shown below in Figure 1. The bibliometric analysis has been done to assess (BIM and Facility management emergence and roll through a systematic literature review. The analysis identified several highly cited articles that have shaped the discourse on BIM) and Facility management.

These include seminal works that address the theoretical frameworks, case studies, and empirical research on the benefits and challenges of integrating BIM and Facility management. This bibliometric analysis is conducted based on the Web of Science (WoS) database holdings from the end of 2018 to 2024. More empirical studies are needed to focus on the long-term impacts of BIM and Facility management adoption on project outcomes and sustainability. More research is required in niche sectors within the construction industry to understand BIM and Facility management’s specific applications and benefits.

The following are some of these publications that touch on the same way of this research^4^. Kim et al. (2020) reported that BIM was highly effective in ensuring that preventive maintenance was conducted on time, which helped prevent equipment and system failures. The research suggested that the integration of BIM and the IoT could significantly improve predictive maintenance capabilities.

Another study by Park et al. (2020) reported that BIM systems aided in the management of maintenance data, including maintenance records and plans. The authors concluded that BIM systems could enhance the efficiency of maintenance management, reduce maintenance costs, and minimize the occurrence of unexpected system failures^5^. Lee et al. (2018) showed that the integration of BIM and facilities management can help stakeholders visualize maintenance tasks, leading to improved communication among stakeholders and reduced costs. The study also highlighted the importance of proper training and education for those involved in BIM implementation^6^. Liu et al. (2019) conducted a systematic review of the literature to examine the barriers and enablers of BIM implementation in the construction industry. The authors identified several key factors that influence BIM implementation, including the lack of standardization across the industry, a lack of expertise among employees, and limited technological infrastructure. The authors suggested that addressing these issues could lead to the wider adoption of BIM systems in the construction industry^7^.

This paper reviewed IoT, which has become a prevalent technology, and its utilization in various industries, including maintenance, which has significantly increased in recent years. IoT-based maintenance systems provide real-time data analysis and monitoring, thus improving the maintenance processes’ effectiveness and efficiency. Chen et al. (2020) analyzed the use of predictive maintenance in various industries, including aviation, manufacturing, and transportation. The study found that the implementation of IoT-based maintenance reduced unplanned downtime and improved the equipment’s lifetime^8^. Alnakhli and Khan (2021) evaluated the holistic IoT-based maintenance system’s effectiveness for the manufacturing industry.

The study revealed that IoT-based maintenance systems can detect equipment faults early and accurately, ensuring maintenance is conducted before any system failures occur, thereby reducing costs and downtime. The authors concluded that IoT-based maintenance systems can replace traditional maintenance methods and improve the management of maintenance data and decision-making^9^. A systematic review conducted by Uhls et al. (2020) aimed at analyzing the effectiveness of IoT-based maintenance systems in the wind energy sector. The study reported that IoT-based maintenance systems enabled continuous tracking of wind turbines and real-time data analysis to identify potential failure points accurately. This results in a decrease in unplanned downtime and an increase in turbine productivity^10^.

Finally, Ma et al. (2020) conducted a study to evaluate the implementation of IoT-based maintenance in data centers. The study reported that IoT-based maintenance systems predict maintenance before equipment failure, reducing unplanned downtime and power consumption. Additionally, IoT-based maintenance can assist in the predictive maintenance processes of power distribution units and cooling systems^11^.

Overall, FMS solutions provide facility management teams with comprehensive tools to manage key components of building operations efficiently. By utilizing a single system for maintenance management, space utilization, energy consumption, compliance, and asset management, facility managers can increase efficiency, reduce costs, maximize occupancy, and create more sustainable operations^12^.

This is important to key stakeholders as it directly impacts costs and profits. The main objective of a maintenance budget system is to ensure that organizations allocate the necessary funds required to maintain their assets, while also minimizing costs and optimizing resource utilization^13^.

**Fig. 2:**
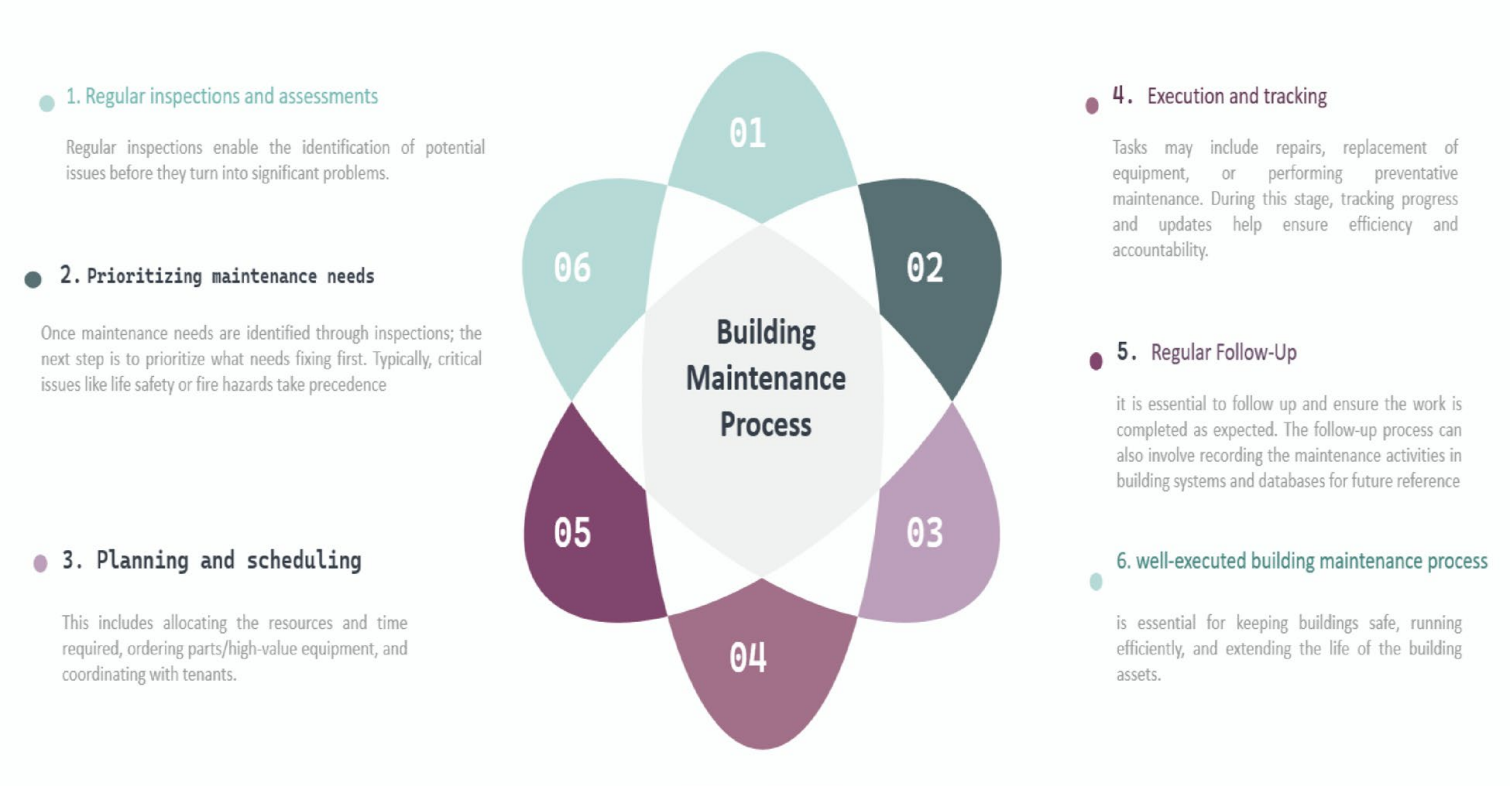
Main maintenance process of building^26^.

By implementing a maintenance budget system, organizations gain greater control over their maintenance expenses, reducing operational risks and ensuring their assets’ long-term sustainability. The building maintenance process typically includes activities and tasks aimed at ensuring upkeep, safety, and efficiency. These activities may vary depending on the size, age, and type of the building and the scope of maintenance required. Figure 2 shows the building maintenance process^14^.

The above bibliometric analysis reveals a dynamic and rapidly evolving literature on BIM-FM in the construction industry. The increasing number of publications and citations reflects the critical role of these technologies in transforming construction practices. Future research should address existing gaps and explore the broader implications of BIM and Facility management integration. Furthermore, the integration of advanced technologies like Artificial Intelligence and the Internet of Things is paving the way for even greater innovations in construction processes. As the industry continues to evolve, ongoing research is essential to address existing gaps and explore the long-term impacts of BIM and Facility management on sustainability and project management.

The assessment framework for BIM and Facility management readiness in the construction industry, presented by^15^ Alnaser et al. (2024), employs structural equation modeling to evaluate the key success factors (KSFs) vital for the adoption of BIM-FM technologies. The study identifies critical KSFs, including organizational readiness, technology availability, and financial investment, emphasizing that sustainability considerations significantly influence BIM-FM deployment. Their findings highlight the necessity for a holistic understanding of the interplay between these factors and sustainable parameters, ultimately aiming to enhance project sustainability.

In the literature review by^16^ Fernández-Mora et al. (2022), the integration of structural projects into the BIM paradigm is examined, revealing that effective collaboration and data sharing across disciplines are essential for maximizing the benefits of BIM. Their analysis underscores the importance of robust modeling techniques and interoperability standards to facilitate better integration of structural engineering within BIM frameworks.

Reference^17^ Bellos et al. (2022) focus on the holistic renovation of a multi-family building in Greece through dynamic simulation analysis. Their research illustrates how integrating advanced simulation tools within the BIM framework can optimize renovation strategies, leading to improved energy efficiency and overall building performance. The study demonstrates the practical applications of BIM in enhancing sustainability outcomes in existing structures. Reference^18^ Carvalho et al. (2021) analyze the feasibility of BIM platforms to support building sustainability assessments. Their findings indicate that while BIM tools offer substantial potential for sustainability evaluation, challenges remain in standardizing metrics and ensuring that platforms are user-friendly for practitioners. The research advocates for the development of comprehensive guidelines to enhance the practical application of BIM in sustainability assessments.

Reference^19^ Okakpu et al. (2018) propose a framework aimed at investigating effective BIM adoption for building refurbishment projects. Their study emphasizes the need for contextual factors, such as regulatory frameworks and the specific conditions of refurbishment projects, to be considered in BIM implementation strategies.

Many BIM-integrated facility management plugins require specialized knowledge, making them inaccessible to non-expert users. To address this gap, the proposed user-friendly plugin simplifies maintenance planning and budgeting, allowing broader accessibility. Additionally, the framework leverages real-time data and predictive models, enhancing maintenance scheduling and resource allocation. Furthermore, current BIM–FM solutions often lack adaptive decision support mechanisms that dynamically optimize maintenance strategies. The proposed plugin provides a flexible and scalable solution, improving overall facility management efficiency.

The proposed framework serves as a guide for practitioners to navigate the complexities of integrating BIM into refurbishment processes effectively. Collectively, these studies illustrate the transformative potential of BIM and FM technologies in promoting sustainability within the construction industry while also highlighting the challenges and necessary conditions for successful adoption and implementation. So, the gap between the previous works and the problem statement can be summarized in the following lines.

As a part of the facility management process, a maintenance system problem statement is a clear and concise statement that describes an issue faced by an organization’s maintenance system. It outlines the challenges and obstacles that the organization is currently facing in its maintenance processes and identifies areas for improvement. Maintenance system problem statements can include experiencing significant downtime and maintenance costs due to an inconsistent and reactive maintenance approach^20^. Equipment failures are not being detected and resolved proactively, leading to costly repairs and lost production time.

Additionally, unused spare inventory consumes valuable space, and maintenance staff struggle to keep up with documentation and reporting requirements. The current maintenance system is inefficient and ineffective, increasing downtime and costs^21,22^. There is a need to establish a reliable and consistent maintenance program that focuses on preventive maintenance, streamlines inventory management, and utilizes technology to improve documentation and reporting. These problem statements highlight the issues that the organization is facing with its current maintenance system, which leads to higher costs, lower productivity, and inefficient use of resources. Existing BIM–FM plugins typically require specialized expertise, making them difficult for non-experts to use^23^. In contrast, the proposed plugin works as a user-friendly interface to enhance FM optimization solution, simplifying facility management and increasing accessibility for a broader range of users. Additionally, unlike traditional solutions, this plugin leverages real-time data and predictive models to enhance maintenance scheduling, ensuring more efficient planning and resource allocation.

## Methodology

**Fig. 3:**
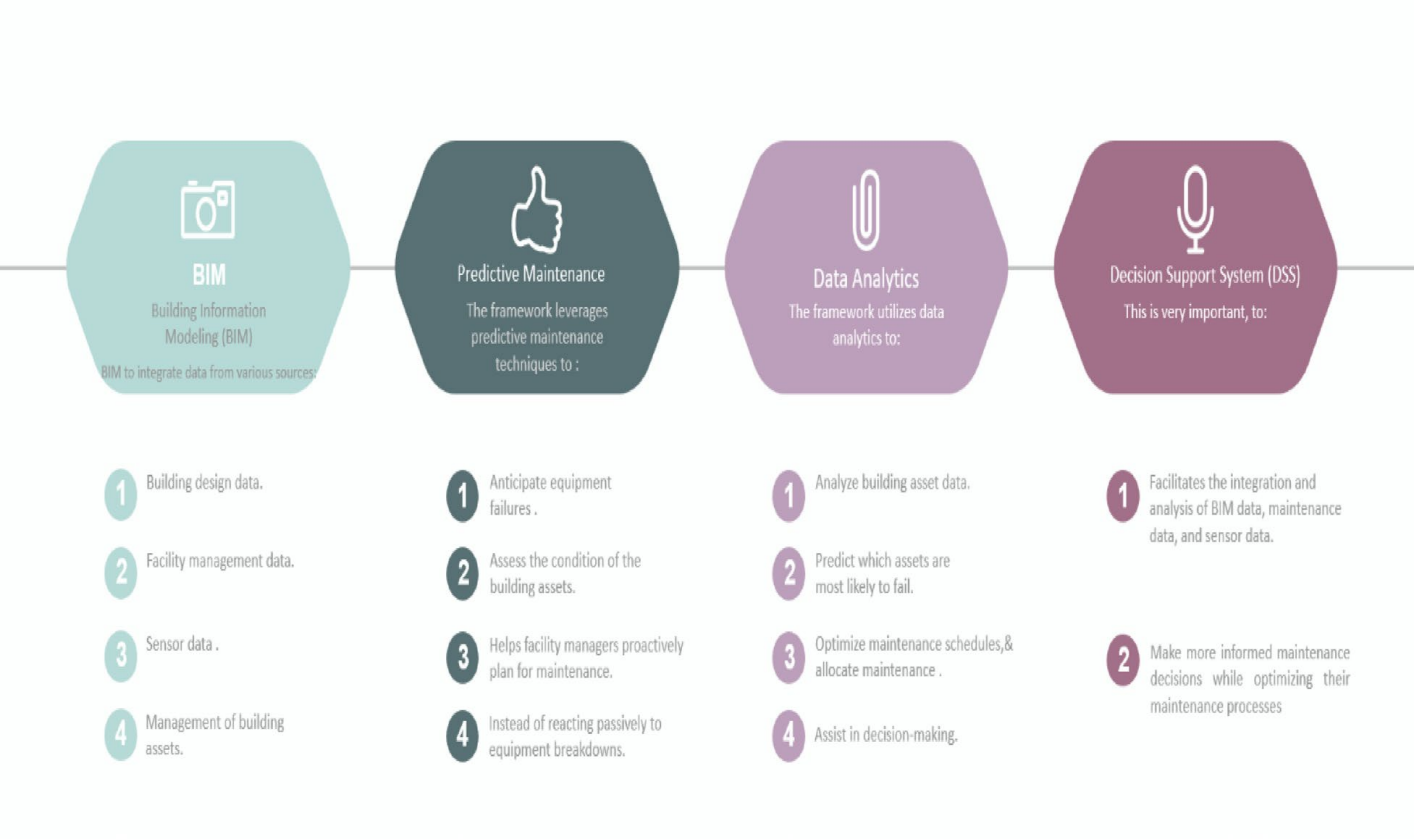
Key components of the framework.

The main goal of this research is to create and implement an innovative yet scalable FM optimum solution without the need for expertise, with an optimal cost, and within an available budget. To achieve this goal, the proposed framework that was created using Python will use real-time data and predictive models to optimize maintenance plans, schedules, and resource allocations. The proposed BIM-based decision support system framework for predictive maintenance management of building facilities is a comprehensive approach to optimizing the maintenance processes of building facilities. The framework is based on the integration of BIM, predictive maintenance, and data analytics to provide building facility managers with the necessary information to make informed maintenance decisions. Figure 3 shows the key components of the framework:

Overall, the proposed BIM-based decision support system framework for predictive maintenance management of building facilities can help facility managers improve their maintenance processes, reduce maintenance downtime, extend the life of the building assets, and reduce maintenance costs. All these problems and others are mentioned in^24,25^, and the proposed system helps to solve most of them. This research suggests that the integration of BIM systems in preventive and predictive maintenance can improve efficiency, reduce costs, and prevent unexpected system failures. However, the success of BIM implementation depends on proper training, standardized guidelines, and addressing technological infrastructure limits.

**Fig. 4:**
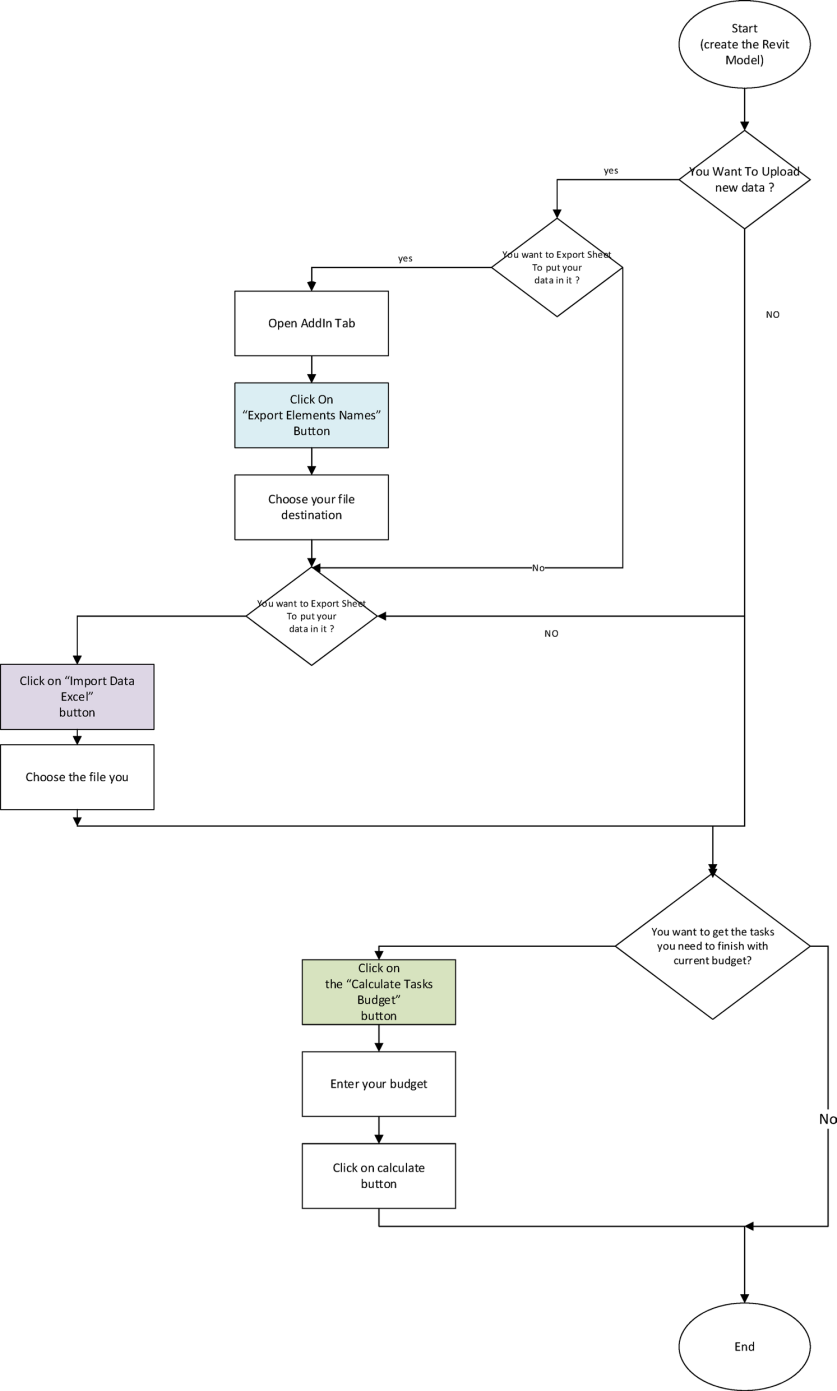
Flowchart for the proposed workflow.

**Fig. 5:**
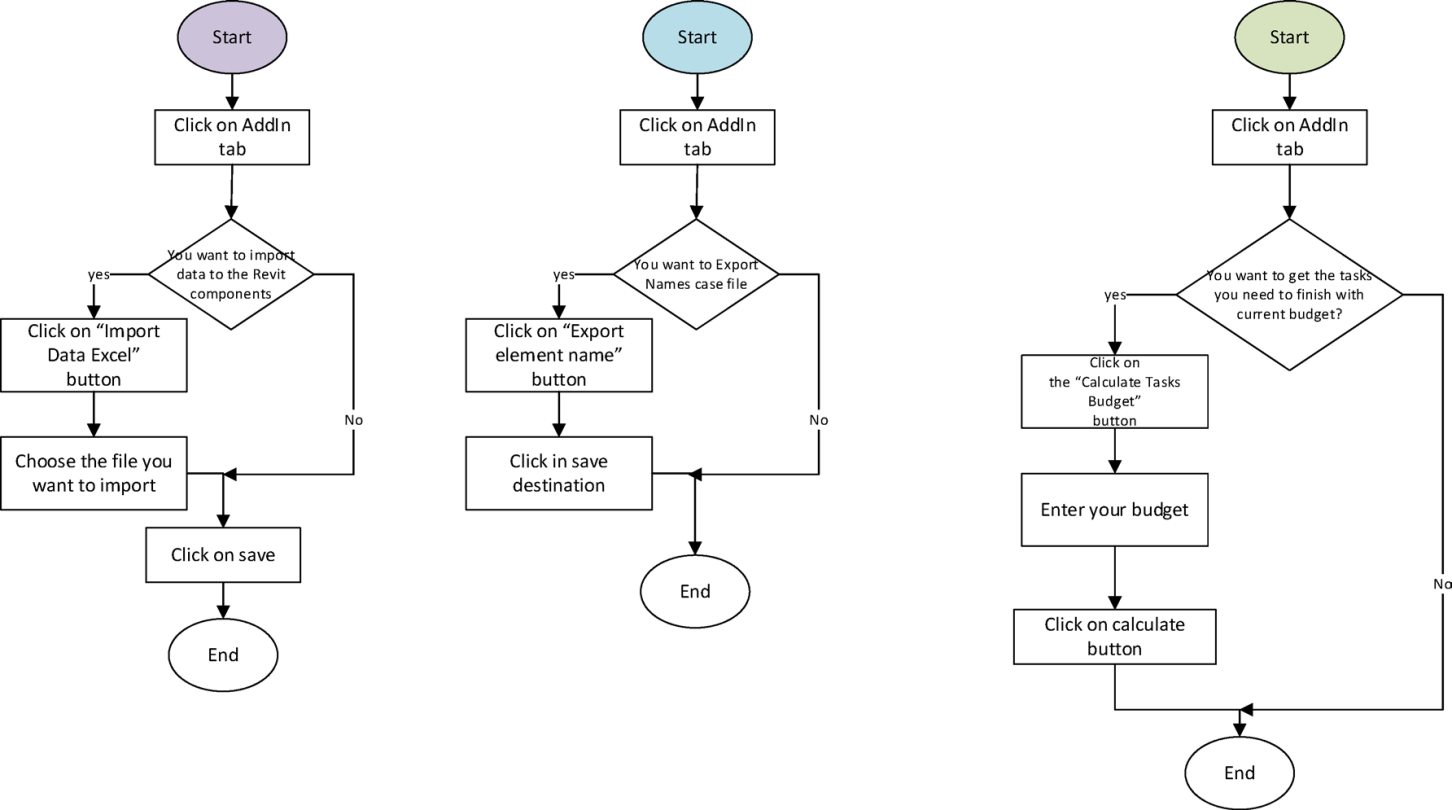
Flowcharts of export elements, import data, and calculation of the budget at the proposed framework.

The flowchart for the proposed workflow is illustrated in Figs. 4 and 5. There are three main steps: (i) creating the model of the building that needs a maintenance system, then exporting elements from Revit into an Excel sheet, (ii) filling the Excel sheet with element data that need to be maintained, and importing data back into Revit, (iii) calculate the budget and create outcome values.

**Fig. 6:**
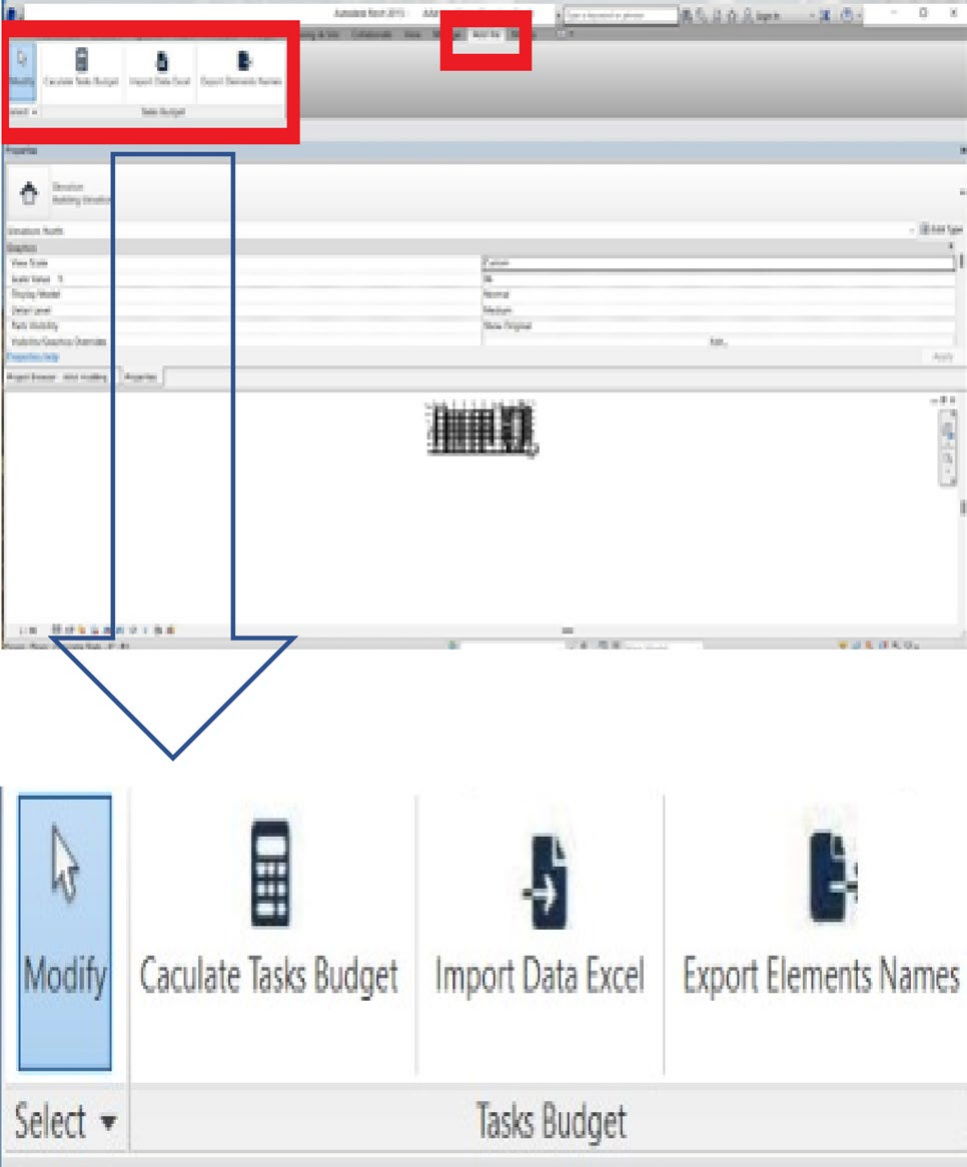
Revit- screen after integrating the proposed add-in.

After adding the add-in to Revit, the screen of Revit changes as shown in Fig. 6, with the three main buttons.

To implement this add-in with Revit, the present work pursued a methodology with the following steps, also documented in Table 1.

**Table 1:** The proposed flowchart and the step-by-step method for its process.

Flowchart	Step by step
	The point of starting the proposed framework
	Creating 3D models in multiple disciplines with a high level of detail (LOD)
	All the components with the data and information of the previous models are now available and ready to share with any tool, like an Excel Sheet.
	This point depends on the technician or responsible engineer with low expertise, just to record on the previous Excel sheet the status of each item and its components.
	After fulfilling the human inspection of the previous Excel sheet, all this information must feed the model by importing it via the proposed system.
	This is the main phase of the proposed workflow, which optimizes all the previous information and the data with some constraints needed, like the allowable budget, the importance index for each item, and the dependency between items and their components.
	The final report (after the algorithm from the previous work) is ready to introduce the optimum solution to the current FM status, and within the allowable budget.
	The end of the proposed workflow.

Firstly, create a BIM (3D, 4D, or 5D) model, which is a digital representation of a building or structure. In the model, different elements like walls, doors, windows, roofs, and floors are used to create the virtual representation of the building. These elements are parametric, meaning they can be easily modified and updated throughout the design process. In the same way, the software not only captures the geometry of the building but also stores important all data associated with each element (Pricing, budgeting, and financial estimations for construction, maintenance, time-related information, including construction sequencing, project timelines, energy efficiency, sustainability metrics, and environmental impact, Maintenance schedules, expected lifespan, and replacement planning). Enhancing a Common Data Environment (CDE) collaboration, real-time modifications by an automated system, strong tracking on time and cost for all elements, risk contingency by reducing over costs, and performance tracking for each item in the mode are the reflections of this data on the proposed system.

Secondly, related to the building information collection, the data collected from the created models includes material properties, dimensions, and structural information. This integration of geometry and data enables better coordination and collaboration among project team members. All of this data is ready to export to an Excel sheet.

Thirdly, the responsible engineer or a maintenance engineer should fill all the cells in the exported Excel sheet, based on their on-site inspection. Column 1 lists the items exported from the software model. Column 2 requires the user to define the importance of each item/facility with a ranking from 1 to 10, where 1 is least important and 10 is most important. Column 3 identifies whether payment for maintaining/replacing this item is immediate (indicated by 1) or can be postponed (indicated by zero). If payment can be postponed, indicate the maximum number of delay days in Column 4. If payment can be deferred, the following information is to be entered: Column 5 is the advanced payment amount, Column 6 is the deferred/remaining payment amount, and Column 7 is the days required to pay the remaining payment amount. Finally, in Column 8, it is important to link the dependencies of each element. This is important information to include and ensures that the user checks all related elements and components. This is typically missed in traditional maintenance schemes.

The third icon in the add-in takes care of the next step, which requires the user to insert the allowable budget for maintenance for this period. Manual documentation ensures data integrity and verification through human oversight, which minimizes the risk of errors or misinterpretations that automated systems may introduce when handling large volumes of data. Additionally, it aids in maintaining compliance with regulatory and industry standards, as many construction projects necessitate specific documentation protocols that automated systems might not fully address. In areas with limited infrastructure or technological capabilities, manual inspections continue to be the most reliable method for accurate data collection and adherence to necessary standards.

The user then runs the algorithm to determine the optimum list of items and their components that need maintenance in this period within the allowable budget. It’s essential to mention that the Multi-Criteria Decision Analysis (MCDA) is the optimization method considered in the proposed plugin. MCDA is used in this study related to the ability of this method to evaluate multiple criteria in decision- making within using qualitative and quantitative evaluation. Finally, a report of the final results is issued. The module helps organizations implement and monitor maintenance plans across multiple assets and provision resources more effectively, reducing costs, increasing asset lifespan, and preventing potential equipment failures or downtime.

The Revit API code implements several optimization techniques for budget allocation and element management. First, it uses (sorting-based optimization) through the Sort-Parameter-Data function to organize elements by dependencies, enabling efficient data retrieval. Second, filtering-based optimization is applied via Filter-Sorted-List-By-Budget, which refines elements based on budget constraints for better resource allocation. Additionally, the code incorporates error handling and validation. Like (Number-Validation-Text-Box), (Check-Needed-Columns), to maintain data integrity before processing. Also, Transaction-based optimization) Ensures efficient modifications by using Revit Transactions, minimizing computational overhead. Finally, memory management optimization) is achieved through memory streams for Excel file handling, enhancing performance during data export and import operations. Together, these techniques improve efficiency, accuracy, and resource management in the Revit environment.

The primary objective of the optimization is to maximize the selection of elements while adhering to a predefined budget constraint. This is implemented through the function called (Filter-Sorted-List-By-Budget), which systematically evaluates and filters elements to ensure compliance with financial limitations. Python is designed to code which used in the proposed system. The following lines show the objective function, which is formulated mathematically as:

1 $$Max\sum\limits_{{i = 1}}^{n}[ {E_{i} } ]$$

Where (E_i) denotes an element satisfying the budget constraint; (n) represents the total number of candidate elements under consideration.

The optimization process is governed by the following parameters:

1. Budget Value—A user-defined financial threshold that constrains element selection.
2. Element IDs & Parameters—Extracted from the Revit model and utilized for filtering and prioritization.
3. Task Importance & Dependencies—Encoded within an Excel file to guide hierarchical processing.
4. Comment Parameter—Serves as a metadata container for element-specific data retrieval.

On the other hand, there are some constraints of the proposed system, which will be summarized below:

### Budget constraint

Ensure the total cost of selected elements does not exceed the specified budget:

2 $$[\sum_{i = 1}^{n} C_i \cdot x_i \leq \text{Budget} ]$$

Where (C_i) = cost of element *I*; (x_i \in {0,1}) indicates whether element *i* is selected (1) or not (0).

### Element selection constraint

Only valid elements from the Revit model (based on parameters or filters) are eligible for inclusion:

3 $$[ x_i = 0 \quad \text{if element } i \text{ does not meet the required filters} ]$$

### Dependency constraint

Enforce ordering or dependencies from your Excel file. For instance, if Task A must be completed before Task B:

4 $$[ x_B \leq x_A ]$$

This prevents selecting Task B without also selecting Task A.

### Task importance priority

You might require certain high-priority elements to be always included or have a minimum representation:

5 $$[ sum_{i \in \text{HighPriority}} x_i \geq k ]$$

Where *k* is a threshold set by the user.

### Comment parameter constraint

Limit selection based on specific metadata tags embedded in the “Comment” parameter:

6 $$[ x_i = 0 \quad \text{if Comment}_i \notin \text{AllowedTags} ].$$

## Validation

**Fig. 7:**
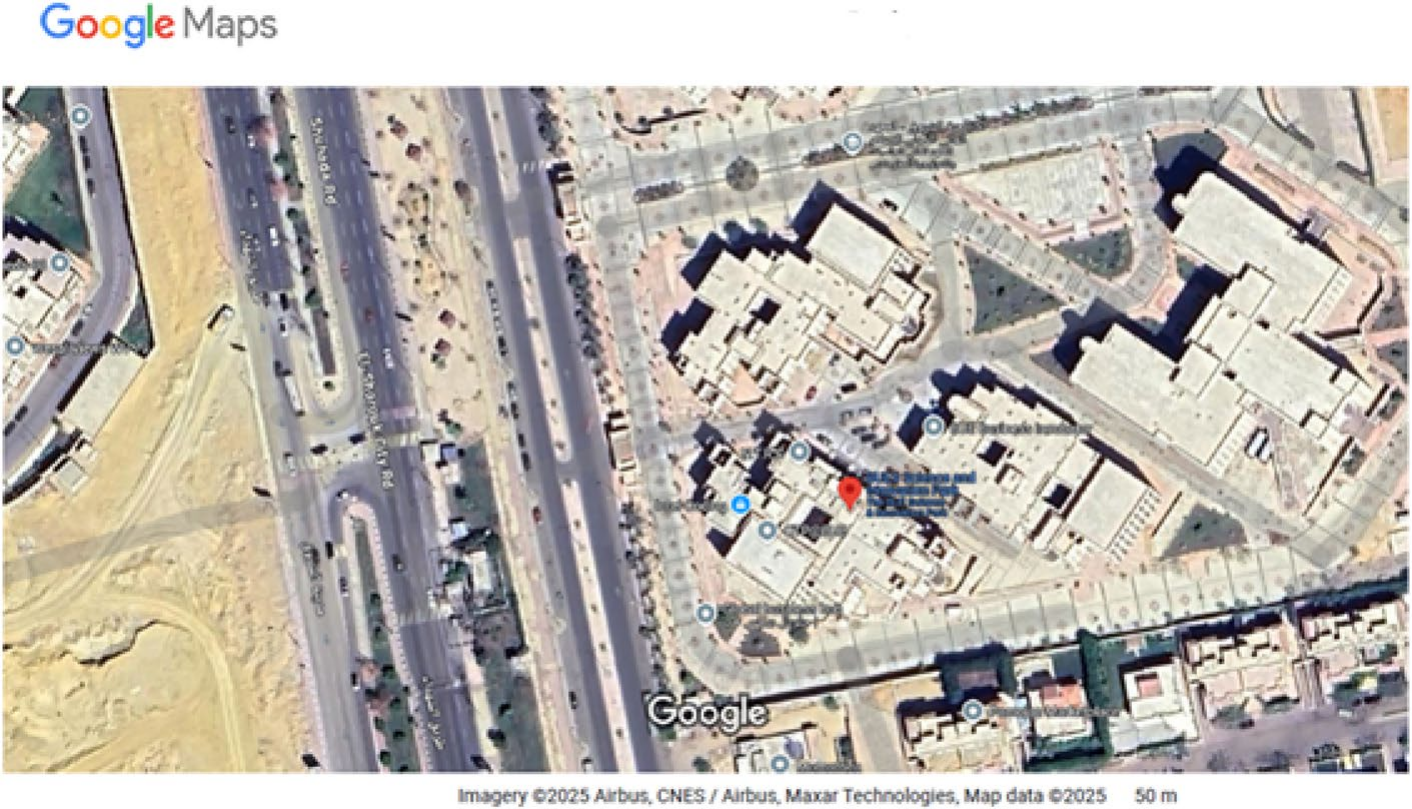
The Location of the actual case study using Google Maps (https://maps.app.goo.gl/AvtggfxrTSjziygb8).

**Fig. 8:**
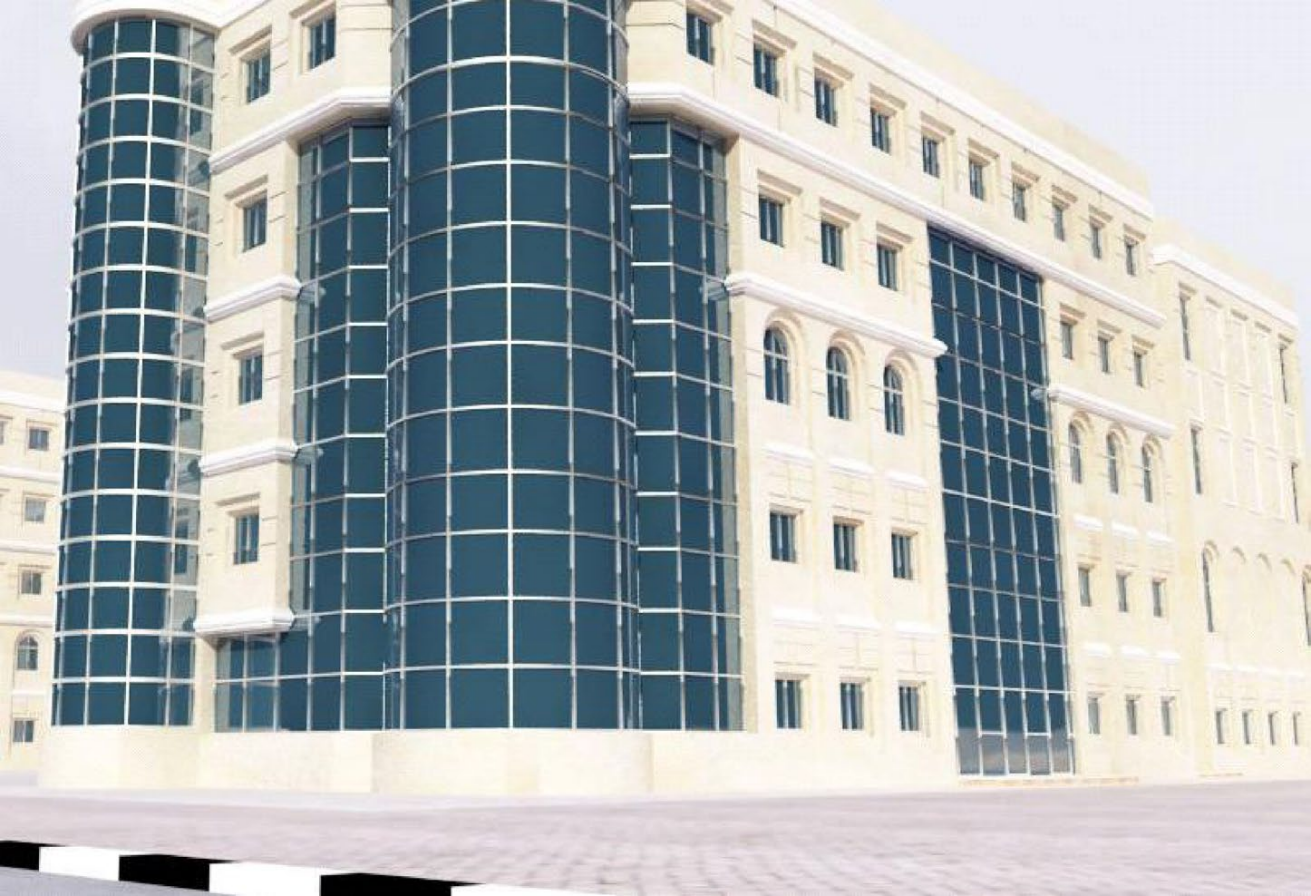
The Center building of the actual case study.

To examine the proposed workflow, a research center building located in El-Shorouk City, Cairo, Egypt, is selected for the study. The workflow and add-in aim to prioritize all building objects and components that require maintenance, based on their importance and relationships with other items. The study focuses solely on building B1, which has a footprint of 2,900 m² per floor. It includes a basement, ground floor, and three typical floors, with a total height of about 16 m, and a perimeter of 215.46 m. The basement parking can accommodate up to 50 cars, as shown in Figs. 7 and 8. The ground floor has a separate entrance and some green areas. The first, second, and third floors are designated for administrative, scientific, and laboratory purposes, respectively. The annual maintenance was performed at the end of July 2022. The estimated budget for maintenance objects and their components using the traditional approach was $450,000, primarily covering civil works, electrical works, MEP, HVAC, and lighting^26^.

**Fig. 9:**
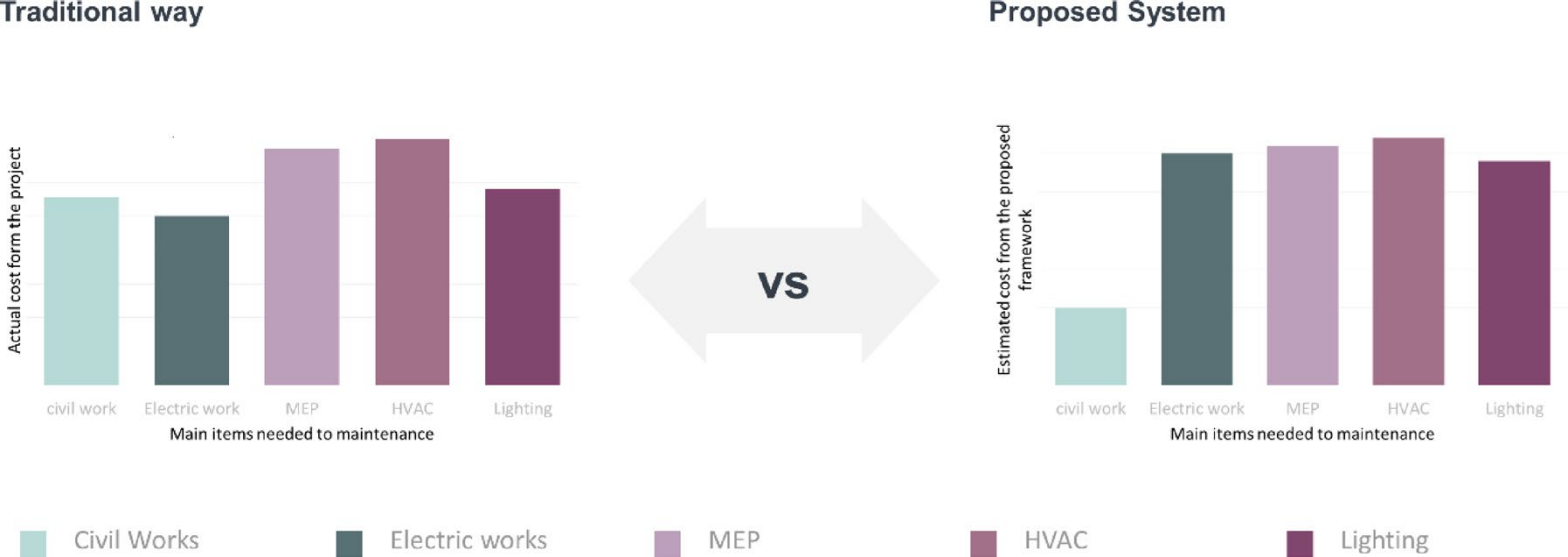
Comparison between the traditional way and the proposed Add-In in the actual case study.

To make a meaningful comparison between the actual case study and the proposed model, numerous iterations were performed for various building components, including civil works, electrical works, MEP, HVAC, and lighting. The main reason for these divisions was the data availability from the actual project. The summary of maintenance costs for each division, from both the actual study and the proposed items, is shown in Fig. 9. The budgeted amount for civil works should be reduced by more than 50%, and the savings in electrical works, MEP, and lighting are approximately 12.55%, 2.21%, and 8.95%, respectively. This analysis demonstrates that using the proposed workflow improves maintenance across items, with significant gains related to the importance index and priority, which is one of the main benefits of the proposed framework. Table 2. Shows a comparison between the traditional way and the proposed system, with the improvement percentages related to the determining criteria.

**Table 2:** Comparison between the traditional way and the proposed system with % of improvement.

Category	Traditional way	Project-based maintenance	Improvement
Data collection	60% manual entry, 40% legacy records	90% automated, 10% validation	+ 50% efficiency
Search time	About 45 min per task (cell-by-cell)	About 10 min (database queries)	About 78% Faster
Error rate	15% (Human /data fragmentation)	5% (standardized workflows)	Decreased by 67% errors
Coverage	70% (focus on urgent repairs)	95% (includes preventive checks)	+ 36% scope
Cost saving	Baseline	30% reduction	Saved/ year

Based on Table 2, project-based methods reduce search time by 78% and errors by 67% through automation. Coverage increases by 36% by integrating civil, MEP, and HVAC systems from project plans. Finally, cost savings amount to 30% annually by avoiding duplicate work.

## Case study, results and discussion

**Fig. 10:**
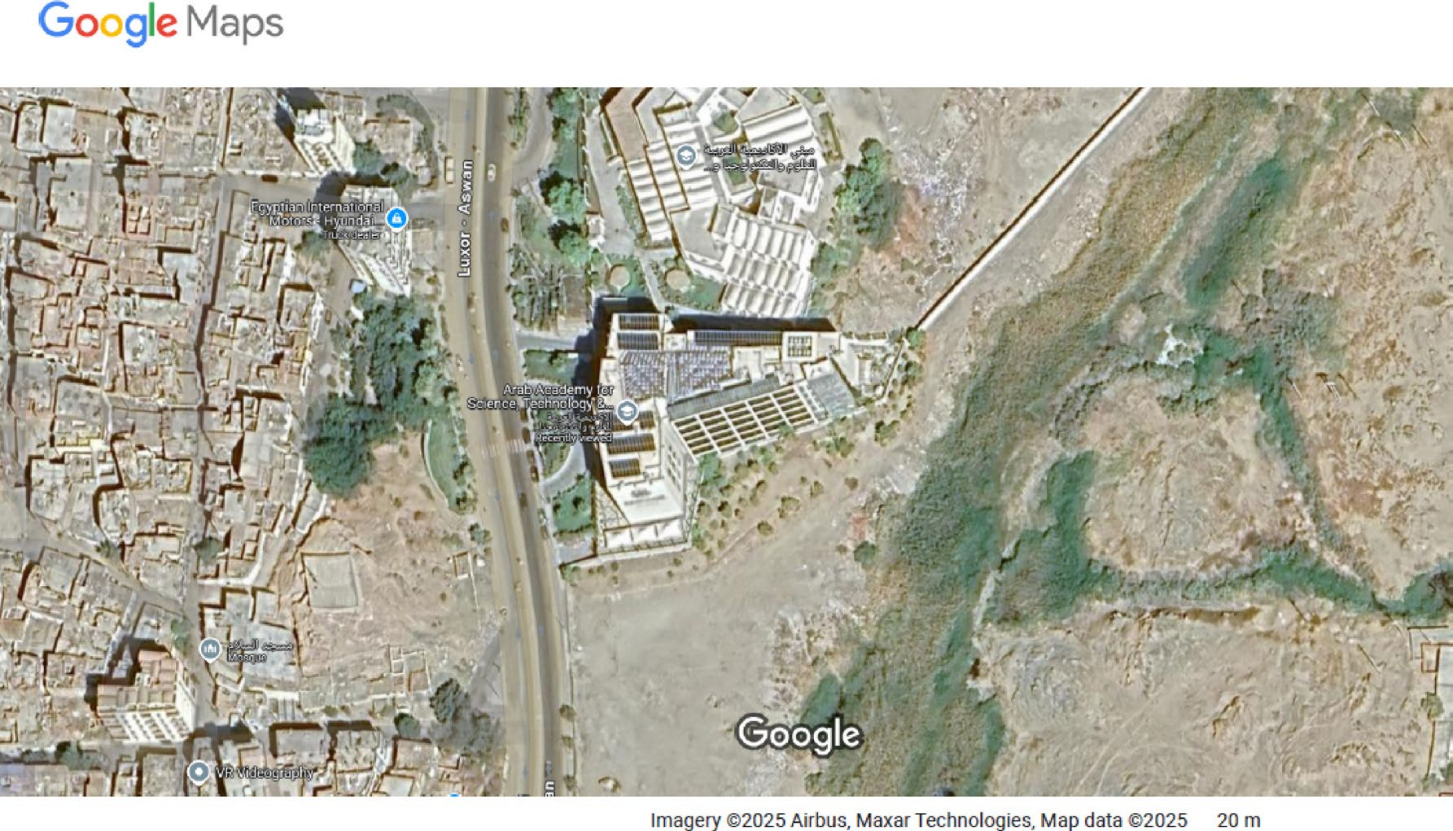
Plane of Building (A) by Google Maps (https://maps.app.goo.gl/5cwpxNrtgHjAicTMA).

**Fig. 11:**
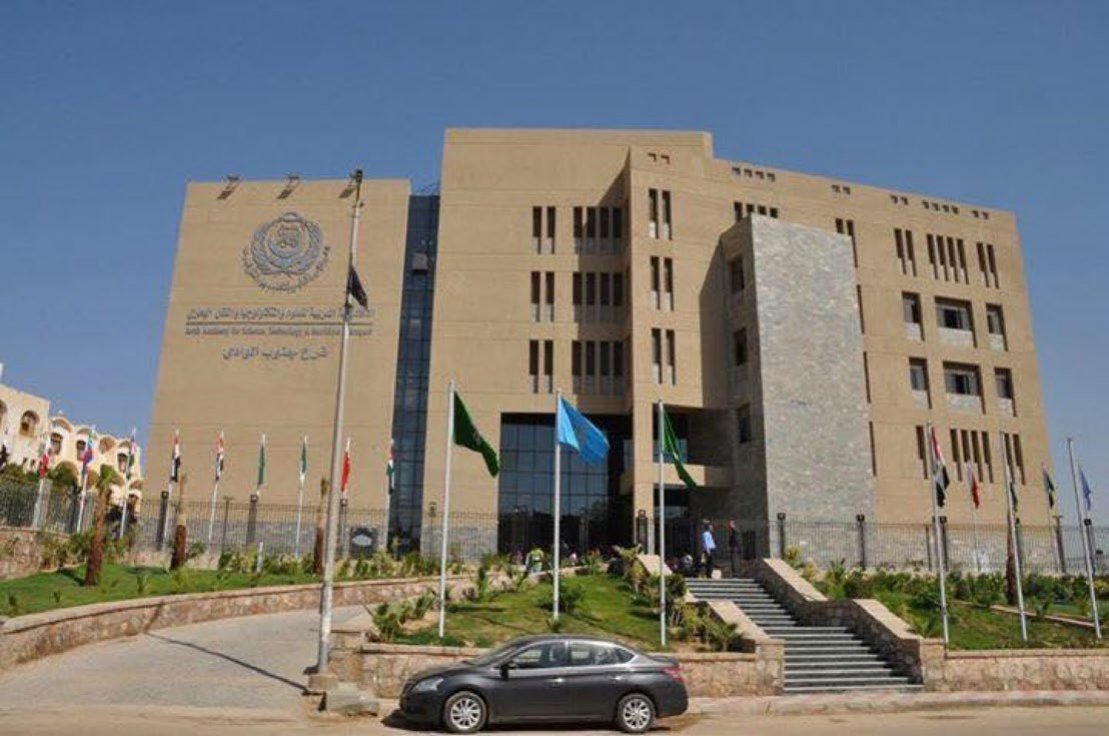
The main faces of building (A).

The case study was carried out to apply the proposed add-in with the BIM model for an actual project located in Egypt. The building is the South Valley branch of the Arab Academy for Science, Technology, and Maritime Transportation (AASTM). The South Valley campus is located at the southern entrance to the city of Aswan. This study is focused on building (A) only. This building consists of a podium with a ground floor and three levels. The land area is 38,000 m^2^ and the built-up area for the Podium and the towers is 197,000 m^2^ as shown in Figs. 10 and 11^24,25^.

**Fig. 12:**
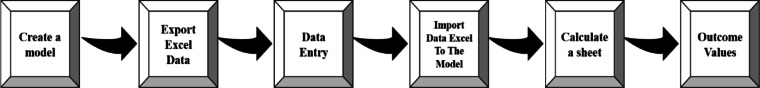
Main process of implementing the add-in on the actual case study.

A database was constructed by collecting data from the engineering team, mainly for the main maintenance works that happened at the end of 2019. The steps to apply this building as shown in the Figure 12 below:

### Create a model

**Fig. 13:**
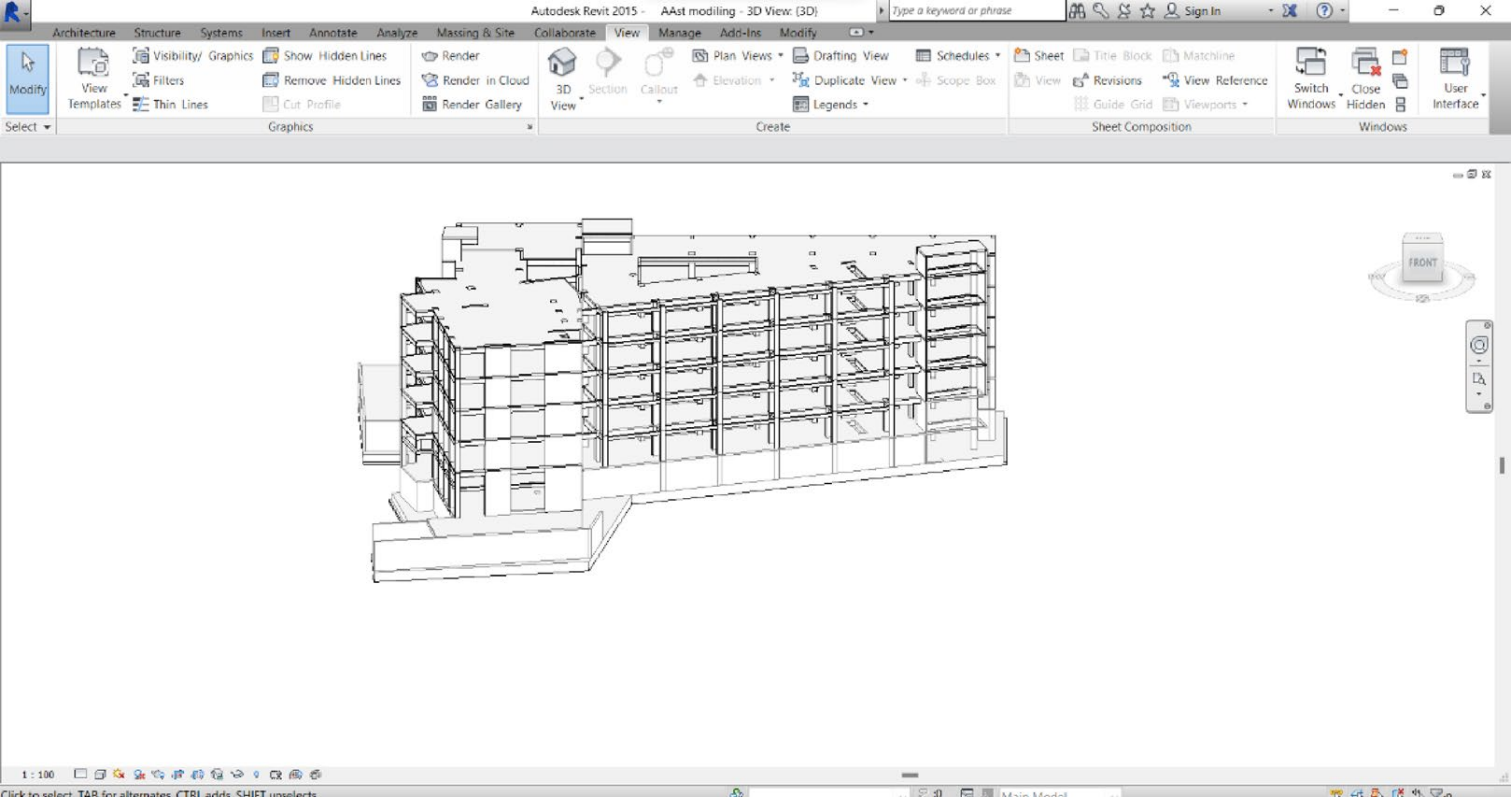
3D Model of the Building Using Revit Software (version 15) https://drive.google.com/file/d/1WTue-ucRZSRHOGheVdMlS-KLYMEzWucB/view?usp=sharing.

This step is very important as it is the basis of all the following steps. In this step, a 3D model is created with a high level of detail (LOD 400) as shown in Fig. 13.

### Export excel data

**Fig. 14:**
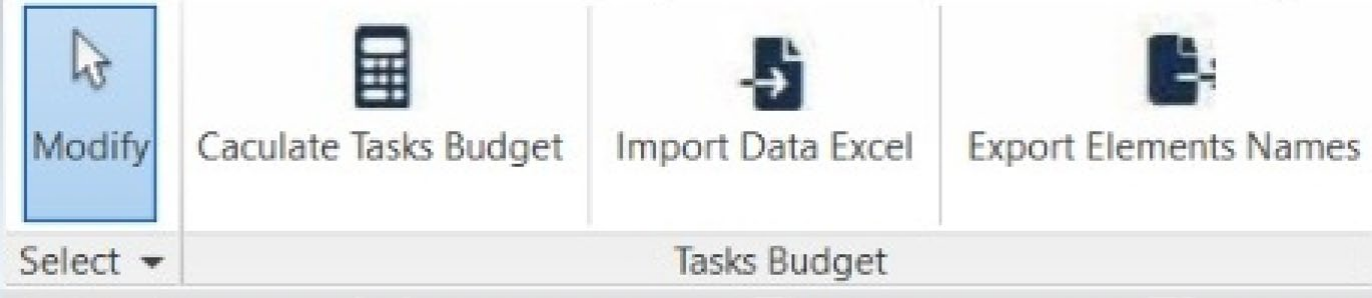
Three main icons at the add-in.

**Fig. 15:**
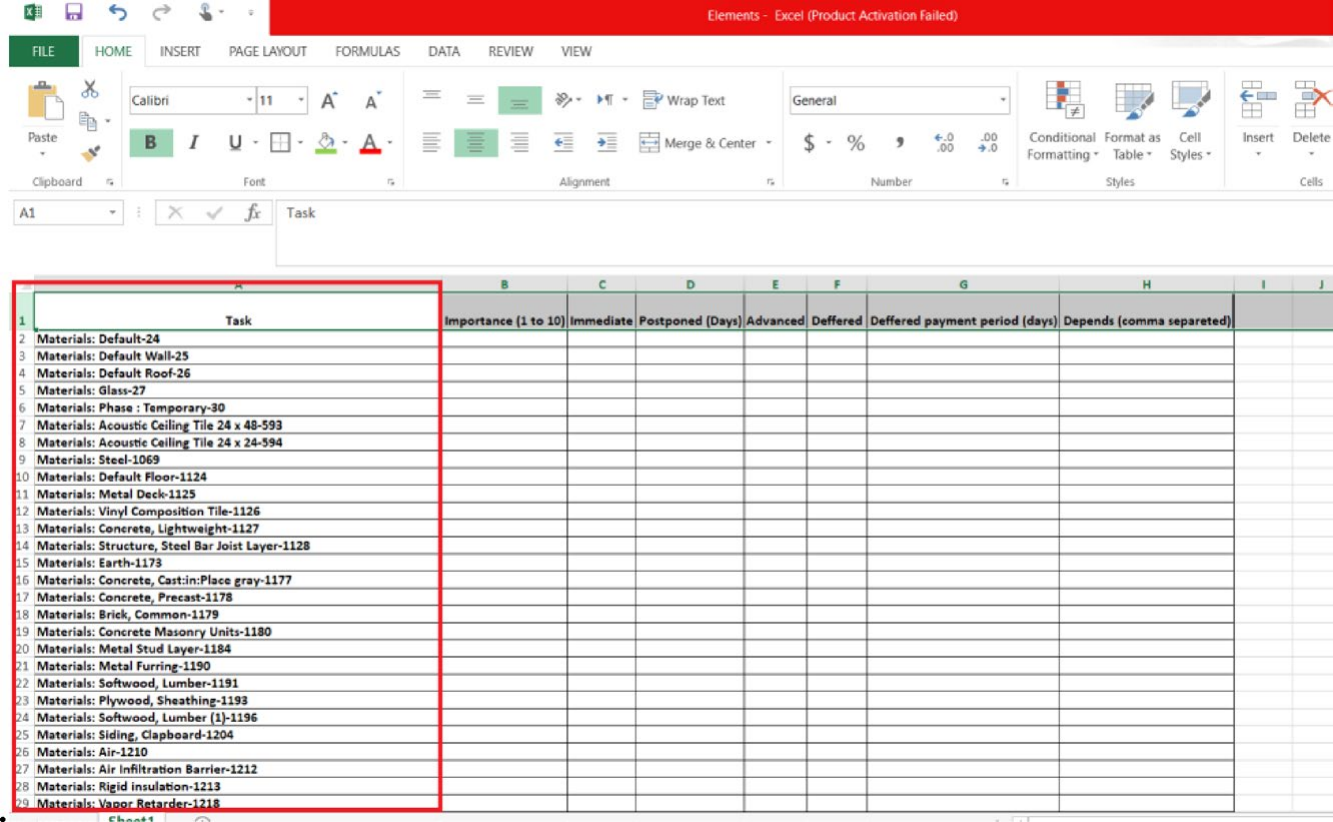
The exported excel sheet.

Once the model is created, all the data for everything drawn in the model is documented and available to use. It should be noted that the user can create Excel sheets for each floor, or area, and not necessarily the entire building. This is a useful tool, as inspections can be done by different engineers at different times. Figure 14 shows the main icons of the add-in and Figure. Figure 15 illustrates a sample of the data for each item drawn from the model (highlighted in the red box).

### Data entry

This step is the main idea of the add-in; at this stage, the user must fill in the remaining cells for the exported Excel sheet from the previous step, as was described in Sect. 4—methodology.

### Import excel data

At this step, the user imports the previous Excel sheet into the 3D model that was created in Revit in step one. Once the Excel sheet is imported into the model, all the data is linked together with a compatible model and is ready for the next and final step.

### Calculate a sheet

**Fig. 16:**
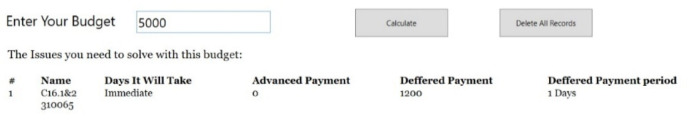
List of maintenance items with allowable budget.

At this step, the add-in asks the user for the total available budget for maintenance at this round, and then the user presses the calculate button. Then, the add-in is running and finds the optimum solution within this budget and the most important objects with their components, as shown in Fig. 16.

**Fig. 17:**
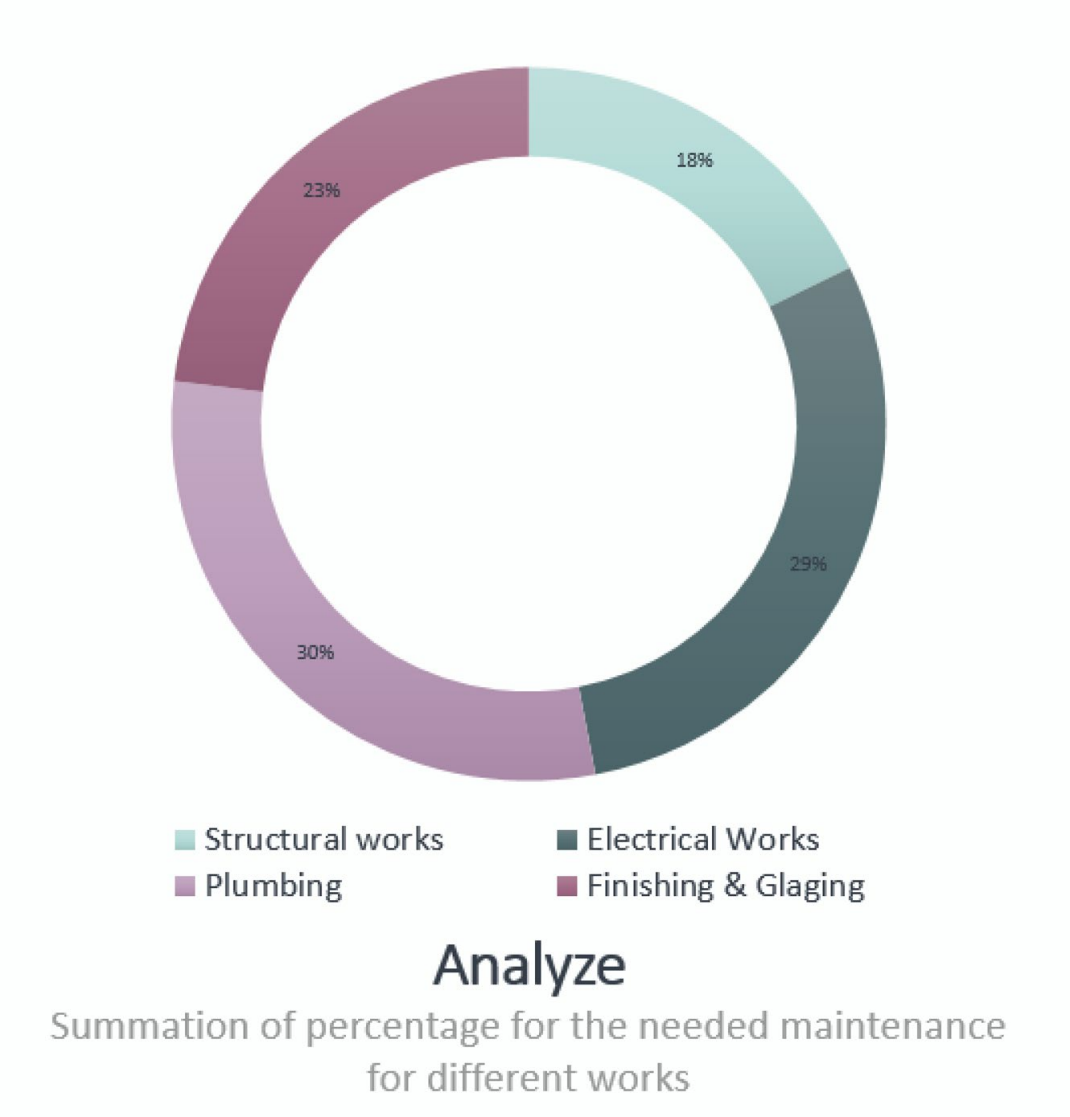
Summary of the budget needed as a percentage for the main items related to the available budget.

Figure 17 below shows the final results of this project as percentages of the total available budget in this period.

As described above, collecting data into BIM models allows for more substantial insight into operations in buildings. The combination of current data with a BIM model can provide the foundation for optimizing facility management and maintenance practices, saving energy and costs, reducing asset downtime, and increasing asset lifespan^27^.

## Conclusions, limitations, and further works

BIM maintenance system plugins help streamline maintenance management processes and improve the overall usability of the software for building operations purposes. The proposed workflow for this research is intended to simplify maintenance processes and optimize budgets for building operations^28^. In conclusion, this research:

- The proposed workflow is very easy to use and accurate, which reflects on the production rates of the decision-makers.
- The optimized maintenance list, which has been introduced as a final report, is robust together and clear to everyone who deals with this research.
- One of the most beneficial aspects of the proposed flowchart is the good linking between each item and its components, which helps to minimize the cost of the maintenance phase.
- The calculations and the validation mentioned about 24% of the budget.
- The optimization solution introduces a final report for the important items, which also includes a small financial statement that helps the company’s financial management.
- The proposed plugin clearly shows decision-making at the maintenance stage.

On the other hand, data collection from the model must be highly detailed. The proposed plugin is suitable only for buildings, and the actual status of the facilities and the inspection phase are the main limitations of this research.

Finally, this study might be expanded to include the following points as further work:

- Improve the proposed module to cover all the construction projects (buildings, highways, and other public works).
- Remove the human inspection to make it more accurate by using any other method for automatic inspections.
- Improve the proposed module by using IOT and sensors to make it smarter and more qualified.
- Create a new system through an integration between the proposed workflow and digital twin (DT).

## Data Availability

“The datasets used and/or analyzed during the current study are available from the corresponding author on reasonable request.”
